# Intact endothelial autophagy is required to maintain vascular lipid homeostasis

**DOI:** 10.1111/acel.12423

**Published:** 2015-11-24

**Authors:** Kumiko Torisu, Krishna K. Singh, Takehiro Torisu, Fina Lovren, Jie Liu, Yi Pan, Adrian Quan, Azza Ramadan, Mohammed Al‐Omran, Natalie Pankova, Shelley R. Boyd, Subodh Verma, Toren Finkel

**Affiliations:** ^1^ Center for Molecular MedicineNational Heart, Lung and Blood InstituteNIH10 Center DriveBethesdaMD 20892USA; ^2^ Division of Cardiac SurgeryKeenan Research Centre for Biomedical SciencesSt. Michael's HospitalUniversity of Toronto30 Bond StreetTorontoON M5B 1W8Canada; ^3^ Division of Vascular SurgeryKeenan Research Centre for Biomedical SciencesSt. Michael's HospitalUniversity of Toronto30 Bond StreetTorontoON M5B 1W8Canada; ^4^ Department of Ophthalmology and Vision SciencesKeenan Research Centre for Biomedical SciencesSt. Michael's HospitalUniversity of Toronto30 Bond StreetTorontoON M5B 1W8Canada

**Keywords:** autophagy, lipids, atherosclerosis, mouse

## Abstract

The physiological role of autophagic flux within the vascular endothelial layer remains poorly understood. Here, we show that in primary endothelial cells, oxidized and native LDL stimulates autophagosome formation. Moreover, by both confocal and electron microscopy, excess native or modified LDL appears to be engulfed within autophagic structures. Transient knockdown of the essential autophagy gene ATG7 resulted in higher levels of intracellular ^125^I‐LDL and oxidized LDL (OxLDL) accumulation, suggesting that in endothelial cells, autophagy may represent an important mechanism to regulate excess, exogenous lipids. The physiological importance of these observations was assessed using mice containing a conditional deletion of ATG7 within the endothelium. Following acute intravenous infusion of fluorescently labeled OxLDL, mice lacking endothelial expression of ATG7 demonstrated prolonged retention of OxLDL within the retinal pigment epithelium (RPE) and choroidal endothelium of the eye. In a chronic model of lipid excess, we analyzed atherosclerotic burden in ApoE^−/−^mice with or without endothelial autophagic flux. The absence of endothelial autophagy markedly increased atherosclerotic burden. Thus, in both an acute and chronic *in vivo* model, endothelial autophagy appears critically important in limiting lipid accumulation within the vessel wall. As such, strategies that stimulate autophagy, or prevent the age‐dependent decline in autophagic flux, might be particularly beneficial in treating atherosclerotic vascular disease.

Cardiovascular disease, driven in part by the accumulation of modified lipids within the vessel wall, represents the leading cause of death in developed nations (Go *et al*., 2013). Considerable attention and study has been directed at the molecular consequences following the subendothelial deposition of lipids. These consequences include the recruitment of inflammatory cells and the subsequent engulfment of this deposited subendothelial lipid by resident macrophages (Chinetti‐Gbaguidi *et al*., 2014; Randolph, 2014). In contrast, relatively little is known regarding the steps proximal to lipid deposition and what regulatory role, if any, the endothelium might play in this process.

While evidence is limited for the case of the endothelium, in other cell types it appears that autophagy may play a critical role in maintaining overall lipid homeostasis. For instance, mice bearing ATG5‐deficient macrophages appear to exhibit increased plaque formation when bred with pro‐atherogenic mouse strains (Liao *et al*., 2012; Razani *et al*., 2012). While the basis for this increased plaque burden is undoubtedly complex, evidence suggests that macrophage‐specific ATG5 deletion results in impaired lipophagy (Sergin & Razani, 2014). Lipophagy refers to the specific degradation of lipids by the autophagic machinery. This concept was perhaps first described in the liver where genetic disruption of macroautophagy led to the accumulation of lipid droplets (Singh *et al*., 2009). These initial observations have been extended and lipophagy now appears to be important in maintaining lipid homeostasis in a diverse set of cell types from neurons to fibroblasts (Singh *et al*., 2009; Liu & Czaja, 2013; Settembre & Ballabio, 2014). Here, we demonstrate an important role for endothelial autophagy in maintaining vascular lipid homeostasis.

It has been previously observed that when endothelial cells in culture are exposed to OxLDL they respond with an increase in autophagosomes (Nowicki *et al*., 2007; Zhang *et al*., 2010; Muller *et al*., 2011; Menghini *et al*., 2014). In general, autophagic flux can be assessed by simultaneously analyzing the levels of LC3‐II, which labels autophagosome membranes, and measuring the levels of p62, a protein cleared via autophagic degradation. The combination of increased LC3‐II along with decreased p62 levels is consistent with an overall increase in autophagic flux. Consistent with past observations, following exposure of human umbilical vein endothelial cells (HUVECs) to OxLDL, the level of LC3‐I to LC3‐II conversion was increased (Fig. 1A,B). As we have previously described (Torisu *et al*., 2013), siRNA ‐mediated knockdown of ATG7 was effective in reducing ATG7 expression in HUVECs (Fig. S1A,B, Supporting information), and endothelial cells with reduced ATG7 expression had markedly impaired capacity to generate LC3‐II before or after OxLDL exposure (Fig. 1A,B). The level of LC3‐II following OxLDL exposure was further enhanced by the presence of the lysosomal inhibitor chloroquine (Fig. 1C). In contrast, levels of p62 were not as markedly affected after OxLDL exposure (Fig. 1A and Fig. S1C) raising the possibility, as previously noted, of alteration in lysosomal function after OxLDL exposure (Jerome, 2010). When primary endothelial cells were electroporated with a plasmid encoding GFP‐LC3 and subsequently exposed to fluorescently labeled OxLDL, we noted an increase in LC3 dot formation, again consistent with an increase in autophagosomes (Fig. S1D,E). Interestingly, the OxLDL within endothelial cells was often surrounded by GFP‐LC3 positive structures whose appearance was consistent with autophagosomes (Fig. 1D). These observations suggested that following uptake of OxLDL, lipids may be directly or indirectly trafficked into autophagosomes. Moreover, given the growing evidence that intracellular lipids can be degraded by lysosomal lipases (Singh & Cuervo, 2012; Liu & Czaja, 2013; Settembre & Ballabio, 2014), intact endothelial autophagy might modulate levels of OxLDL accumulation. Consistent with this hypothesis, OxLDL accumulated at higher levels in endothelial cells lacking an intact autophagy machinery (Fig. 1E).

**Figure 1:**
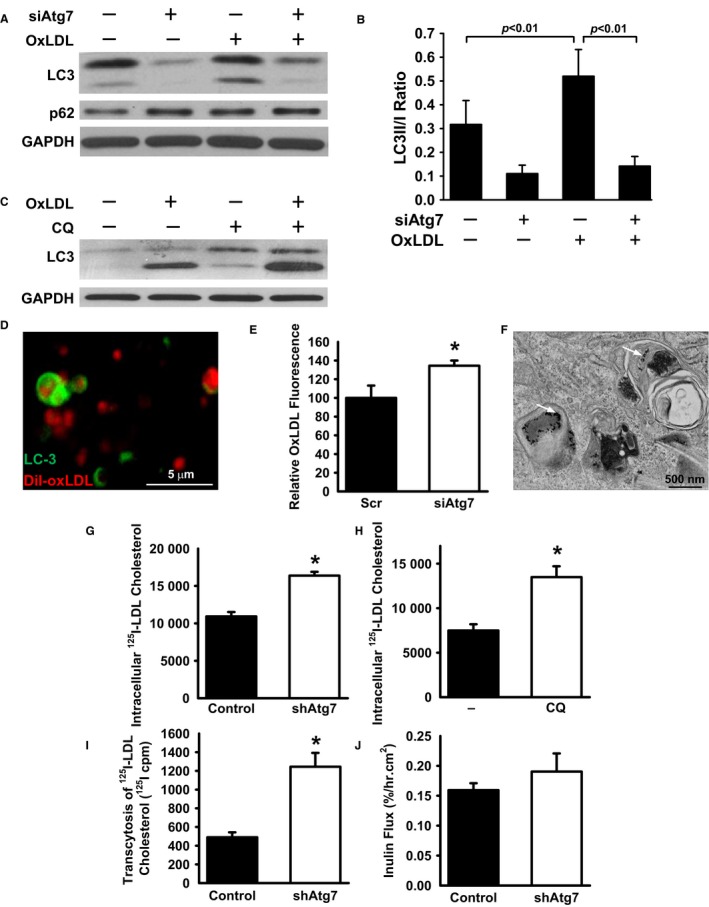
Role of autophagy in endothelial lipid homeostasis. (A) Western blot analysis of HUVECs exposed to OxLDL (50 μg mL^−1^ for 24 h) that were knocked down with either a scrambled RNAi (−) or an RNAi targeting the essential autophagy gene ATG7 (siAtg7). Shown are levels of LC3, where the top band represents LC3‐I and the bottom band LC3‐II, p62, and GAPDH as a loading control. (B) Quantification of the LC3‐II/LC3‐I ratio as a marker of autophagosome formation (*n* = 4–6 blots per group; one‐way ANOVA followed by Bonferroni post hoc test). (C) Levels of LC3‐I and LC3‐II in HUVECs in the presence or absence of OxLDL and the lysosomal inhibitor chloroquine (CQ). (D) Confocal image of HUVECs transfected with a GFP‐LC3 plasmid (green) and exposed to fluorescently labeled OxLDL (red). The lipid, in some cases, appears to be surrounded by circular LC‐3‐coated structures, consistent with an autophagosome. (E) Accumulation of OxLDL in endothelial cells transfected with a scrambled siRNA or one targeting ATG7 (*n* = 5 per condition, *P < 0.05). (F) Electron micrographs of HUVECs incubated with native LDL that had been coupled to gold beads. Micrographs demonstrate gold beads within autophagosomes (white arrows). (G) Intracellular‐labeled ^125^I‐LDL accumulation in control knocked down cells or in cells knocked down for ATG7. (*n* = 3, *P < 0.01 by two‐tailed unpaired t‐test with Welch's correction, representative of four similar independent experiments). (H) Intracellular ^125^I‐LDL accumulation in un‐infected HUVECs following treatment with chloroquine (CQ;* n* = 4, *P < 0.01 by two‐tailed unpaired t‐test with Welch's correction, representative of three similar independent experiments). (I) Level of *in vitro* transcytosis of labeled ^125^I‐LDL across HUVEC monolayer cultures following control or ATG7 knockdown. (*n* = 3, *P < 0.01 by two‐tailed unpaired t‐test with Welch's correction, representative of four similar independent experiments. (J) Levels of fluorescently tagged inulin flux through HUVEC monolayer cultures. (*n* = 3, *P* = NS).

Native LDL is thought to be taken up by receptor‐mediated endocytosis and delivered to lysosomes through the endosomal pathway. It has been appreciated for some time that genetic deficiencies in lysosomal lipase impairs cholesterol hydrolysis leading to intracellular lipid accumulation (Goldstein *et al*., 1975). Increasing evidence also suggests that endosomal and autophagosomal pathways are closely connected and share common machinery (Rusten *et al*., 2012; Hyttinen *et al*., 2013). Electron micrographs of HUVECs incubated with gold‐labeled native LDL identified gold‐labeled particles within double membrane autophagosomal structures (Fig. 1F). Similar to what we observed following OxLDL treatment, native LDL also appeared to increase the number of autophagosomes (Fig. S1F,G) and to be in close contact with GFP‐LC3 positive structures (Fig. S1H). As we have previously described, we next used lentivirus‐mediated shRNAs to stably knockdown ATG7 expression (Torisu *et al*., 2013). We noted that ^125^I‐LDL cholesterol accumulated to a higher level in cells with reduced Atg7 (Fig. 1G). Increased ^125^I‐LDL cholesterol accumulation was also observed following treatment of endothelial cells with the autophagy and lysosomal inhibitor chloroquine (Fig. 1H). This increase was not due to any apparent alterations in levels of the LDL receptor of surface binding of ^125^I‐LDL cholesterol (Fig. S1I,J).

The deposition of native or modified lipids in the sub‐endothelial space is thought to contribute to vascular disease. To begin to model this phenomenon *in vitro*, we analyzed the transcytosis of ^125^I‐LDL cholesterol in confluent monolayers of HUVECs following control or ATG7 knockdown. As noted in Fig. 1I, following a 24‐h incubation, there was evidence for increased ^125^I‐LDL transcytosis across ATG7‐deficient endothelial monolayers. In contrast, Atg7 knockdown did not affect the permeability to inulin (Fig. 1J). The differences in transcytosis rates may simply reflect higher rates of accumulation of ^125^I‐LDL cholesterol in ATG7 knockdown cells (Fig. 1G) or perhaps may reflect recent observations directly linking autophagy to barrier function (Nighot *et al*., 2015).

While a number of sophisticated *in vitro* assays have been developed for assessing endothelial barrier function (Wegener & Seebach, 2014), we sought to assess a potentially more physiologically relevant model by analyzing mice with a conditional endothelial deletion of ATG7 (Torisu *et al*., 2013). We took advantage of the rich vascular plexus in the eye and the previously described transport of lipids from the choroidal blood vessels to the retinal pigment epithelium (RPE) (Picard *et al*., 2010). To assess whether the acute handling of modified lipids depends on intact endothelial autophagy, we injected control mice (WT/WT; VE‐Cadherin Cre) or Atg7^endo^ mice (fl/fl;VE‐Cadherin Cre) mice with fluorescently labeled OxLDL (dil‐OxLDL).We next analyzed the retinas of mice 24 and 48 h after OxLDL injection by confocal microscopy. Deposits of dil‐OxLDL were evident in the mouse retinal pigment epithelium (RPE) and the choroidal endothelium beneath the RPE. Twenty‐four hours post injection, dil‐OxLDL uptake in the mouse eye was similar in *Atg7* ^endo^ and control mice (data not shown). However, 48 h after dil‐OxLDL administration, the fluorescent tagged dil‐OxLDL was retained at higher levels within the RPE and choroidal endothelium of the *Atg7* ^endo^ mice than in wild‐type tissue (Fig. 2A,B). Quantification of dil‐OxLDL fluorescence confirmed the increased persistence of dil‐OxLDL in *Atg7* ^endo^ mice (Fig. 2C, *n* = 6 eyes per group, *P *< 0.0001 by two‐tailed *t*‐test). Thus, in this acute model of modified LDL exposure, autophagy‐deficient mice demonstrate increased and persistent lipid accumulation.

**Figure 2:**
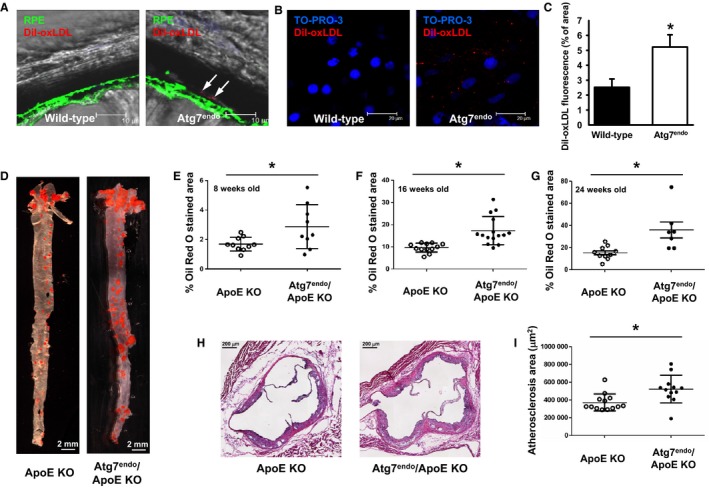
Autophagy regulates *in vivo* vascular lipid deposition. (A) Deposition of fluorescently labeled OxLDL in the retina of control (WT/WT; VE‐cadherin Cre) or Atg7^endo^ mice 48 h after injection. Arrows represent retained diI‐OxLDL particles deposited sub‐RPE, at the level of the RPE basal membrane. (B) Representative whole mount images from Atg7^endo^ mice demonstrating accumulation of fluorescent particles (presumptive vesicles) within binucleated RPE cells and in the adjacent extracellular matrix and endothelium of the choriocapillaris. (C) Quantification of labeled OxLDL in the retina of control or Atg7^endo^ mice (*n* = 6 eyes per group) 48 h after infusion, *P < 0.05 by two‐tailed unpaired *t*‐test. (D) Representative Oil Red O stained aortas from control (WT/WT VE‐cadherin Cre/ApoE^−/−^ abbreviated as ApoE KO) or Atg7^endo^/ApoE KO (fl/fl VE‐Cadherin Cre/ApoE^−/−^) mice. (E) Quantification of Oil Red O staining at 8 weeks (*n* = 10 ApoE KO and *n* = 9 Atg7^endo^/ApoE KO mice, *P < 0.05 two‐tailed unpaired t‐test with Welch's correction). (F) 16 weeks (*n* = 12 ApoE KO and *n* = 15 Atg7^endo^ApoE KO mice, *P < 0.01 by two‐tailed unpaired t‐test with Welch's correction) and (G) 24 weeks of age (*n* = 10 ApoE KO and *n* = 7 Atg7^endo^/ApoE KO mice, *P < 0.03 by two‐tailed unpaired *t*‐test with Welch's correction). (H) Micrographs of the aortic root at 16 weeks of age. (I) Quantification of plaque area at 16 weeks between the two genotypes (*n* = 13 ApoE KO and *n* = 12 Atg7^endo^/ApoEKO mice, *P < 0.01 by two‐tailed unpaired *t*‐test with Welch's correction).

We next sought to assess a more chronic model of lipid exposure by crossing our control and Atg7^endo^ mice into an ApoE^−/−^ background. The presence or absence of endothelial ATG7 did not significantly alter serum lipid or glucose levels, nor did it alter overall body weight or body composition (Table S1, Supporting information). In contrast, consistent with altered lipid handling, we observed increased en face Oil Red O‐positive staining in high‐fat‐fed ApoE^−/−^Atg7^endo^ mice (Fig. 2D). These differences were maintained when mice were analyzed at 8 weeks of age (e.g., 4 weeks of high‐fat diet), 16 weeks of age (12 weeks of high‐fat diet) or 24 weeks of age (20 weeks of high‐fat diet; Fig. 2E–G). A similar analysis of cross‐sectional plaque area in the aortic root was also consistent with the observation that high‐fat‐fed ApoE^−/−^Atg7^endo^ mice had increased atherosclerotic lesion size when compared with control animals (Fig. 2H,I). Preliminary analysis of the plaque composition revealed a trend for increased necrotic area in the ApoE^−/−^Atg7^endo^ mice (Fig. S2, Supporting information).

Taken together, our results demonstrate an important role for endothelial autophagy in maintaining vascular lipid homeostasis. Further work is needed to better define how autophagy modulates the intracellular fate of engulfed lipids (Fig. S1K). Nonetheless, the ability of endothelial cells to target excessive native or modified lipids for autophagy‐dependent degradation appears to be an important mechanism to limit atherosclerotic plaque formation. Our *in vitro* results suggest that knockdown of ATG7 or treatment with chloroquine can increase endothelial lipid accumulation. Given that chloroquine works as an inhibitor of lysosomal processes, the most likely explanation is that similar to what has been described in other tissues; endothelial cells rely, at least in part, on autophagosomal‐mediated delivery of lipids to the lysosome for degradation. In this context, it may be relevant that the gene lysosomal acid lipase A (LIPA) has been recently identified in genome‐wide studies as a potential susceptibility locus for atherosclerotic disease (Wild *et al*., 2011; Vargas‐Alarcon *et al*., 2013). In addition, it is of potential interest that a number of genes associated with lipid metabolism have also been linked to age‐related retinal diseases (Black & Clark, 2015).

Given that autophagic flux is believed to decline as a function of age (Cuervo, 2008), the age‐dependent decline in endothelial autophagy might contribute to the sharp rise in age‐related cardiovascular disease. Interestingly, pharmacological efforts that augment autophagy appear to reverse some of the properties of mouse and human arterial aging (LaRocca *et al*., 2012). Previous studies have also suggested that the atherosclerotic plaque is enriched for autophagosomes (Martinet & De Meyer, 2009). In addition, recent mouse genetic studies have implicated the role of autophagy in macrophage foam cell formation (Muller *et al*., 2011; Ouimet *et al*., 2011; Le Guezennec *et al*., 2012; Razani *et al*., 2012; Sergin & Razani, 2014). Our results complement these observations and suggest that similar to what was observed in macrophages, endothelial autophagy is important in limiting atherosclerotic progression. It is tempting to speculate that enhancing autophagy may therefore be a beneficial strategy to reduce the rate of age‐dependent cardiovascular disease. Such speculation is supported by previous observations including that inhibition of mTOR (a kinase known to function as a negative regulator of autophagy) with pharmacological agents such as rapamaycin appears to inhibit atherosclerosis in a number of different animal models (Castro *et al*., 2004; Pakala *et al*., 2005; Mueller *et al*., 2008). Similarly, strategies such as calorie restriction, that are known to elevate autophagic flux, appear to reduce cardiovascular disease in both mice and humans (Guo *et al*., 2002; Fontana *et al*., 2004). Manipulation of vascular autophagy might therefore be an attractive therapeutic target to potentially limit atherosclerotic disease independent of serum lipid values.

## Supporting information


**Fig. S1.** Characterization of the role of autophagy in endothelial lipid homeostasis.
**Fig. S2.** The role of autophagy in ApoE KO mouse atherosclerotic plaque formation.
**Table S1.** Serum chemistry and body weight.

**Data S1.** Experimental procedures.
