# Supplementary material for: Intact endothelial autophagy is required to maintain vascular lipid homeostasis
Source: Aging Cell. 2015 Nov 24;15(1):187–91. doi: 10.1111/acel.12423 (PMC4717267; doi:10.1111/acel.12423)
Supplement: Supplementary file 1 — Fig. S1. Characterization of the role of autophagy in endothelial lipid homeostasis. Fig. S2. The role of autophagy in ApoE KO mouse atherosclerotic plaque formation. Table S1. Serum chemistry and body weight. [file ACEL-15-187-s001.pdf]

Supplementary Figure 1

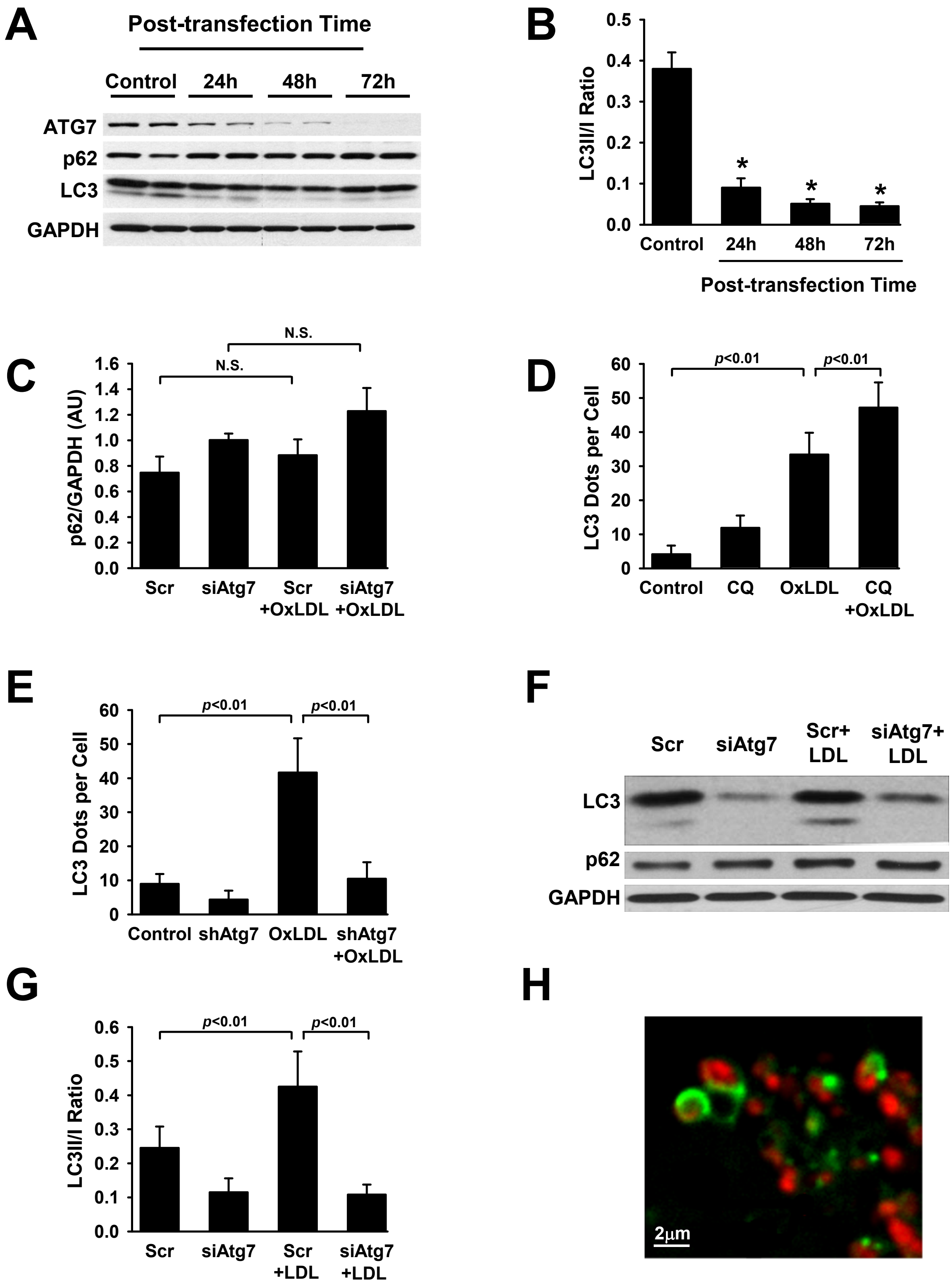

I

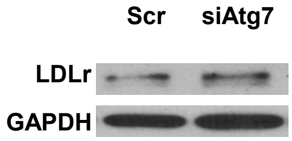
Scr
siAtg7
LDLr
GAPDH

J

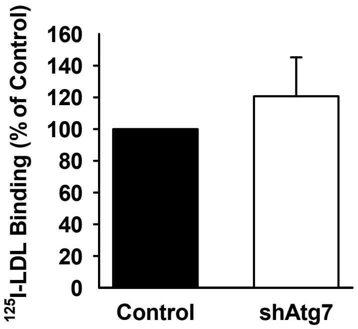
125I-LDL Binding (% of Control)
160
140
120
100
80
60
40
20
0
Control
shAtg7

K

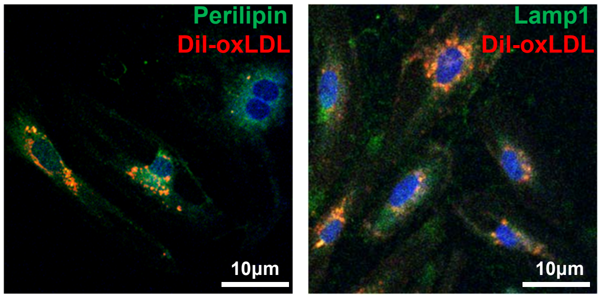
Perilipin
DiI-oxLDL
10µm
Lamp1
DiI-oxLDL
10µm

Supplementary Figure 2

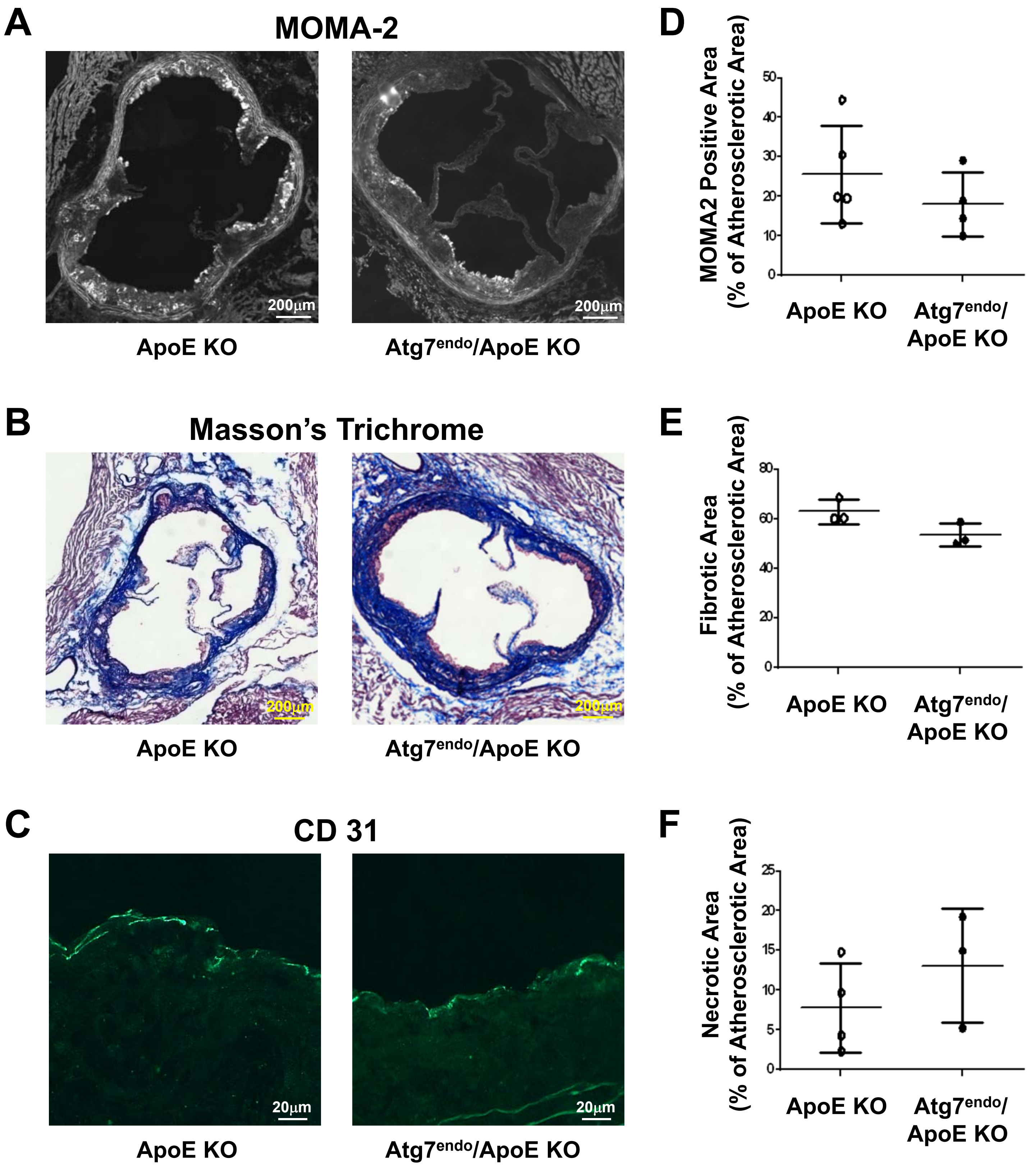

Supplementary Table: Serum chemistry and body weight

	ApoE KO		Atg7^{endo}/ApoE KO		*p*
Total Cholesterol (mg/dl)	563.8 ± 99.4	n=6	525.9 ± 72.2	n=4	0.56
LDL (mg/dl)	194.3 ± 29.9	n=6	194.2 ± 54.2	n=4	0.99
HDL (mg/dl)	11.4 ± 7.2	n=6	9.8 ± 2.9	n=4	0.66
Triacylglycerol (mg/dl)	147.2 ± 51.4	n=6	119.4 ± 27.6	n=4	0.34
Free Fatty Acids (mEq/l)	1.12 ± 0.1	n=11	1.26 ± 0.1	n=11	0.43
Fasting Glucose (mg/dl)	111.7 ± 8.3	n=9	130.5 ± 9.2	n=9	0.15
Body Weight (g)	29.2 ± 1.0	n=9	28.4 ± 2.0	n=9	0.75
Fat Composition (%)	24.8 ± 4.3	n=9	24.1 ± 3.9	n=9	0.91
