# Supplementary material for: Intact endothelial autophagy is required to maintain vascular lipid homeostasis
Source: Aging Cell. 2015 Nov 24;15(1):187–91. doi: 10.1111/acel.12423 (PMC4717267; doi:10.1111/acel.12423)
Supplement: Supplementary file 2 — Data S1. Experimental procedures. [file ACEL-15-187-s002.docx]

**Experimental Procedures:**

**Mice and Cells:**

Atg7^flox/flox^ mice have been previously described ([Komatsu *et al.* 2005](#_ENREF_1)) and were crossed with VE-cadherin Cre mice (Jackson Laboratory) to generate mice with a conditional deletion of ATG7 within endothelial cells (Atg7^endo^) as previously described ([Torisu *et al.* 2013](#_ENREF_5)). All experiments were approved by Animal Care and Use Program at the NIH and/or in accordance with the guidelines of the Canadian Council on Animal Care and approved by the St. Michael's Hospital Animal Care Committee.

Pooled primary human umbilical vein endothelial cells (HUVECs) were grown as previously described ([Torisu *et al.* 2013](#_ENREF_5)). For pharmacological inhibition of autophagy, HUVECs were treated with 25 µM chloroquine (CQ; Sigma) for 16 hours. For live cell imaging, HUVECs were plated onto 35 mm glass bottomed culture dishes (MatTek). Human low density lipoprotein and human DiI-oxidized low density lipoprotein was purchased from Kalen Biotechnologies. Labeled Iodine-125 LDL cholesterol was obtained from PerkinElmer**.**

**Plasmids and Lentiviral vectors:**

The GFP-LC3 plasmids have been previously described ([Lee *et al.* 2008](#_ENREF_2)). For siRNA-mediated knockdown of ATG7, ATG7 siRNA or a scrambled control siRNA (Ambion) was used ([Singh *et al.* 2015](#_ENREF_4)). In brief, HUVECs were transfected with 2 nmol/L of the indicated siRNA using the Dharmafect transfection reagent (Dharmacon). Lentiviral vector for stable shRNA mediated knockdown of ATG7 was purchased from Open Biosystems. Amplification of lentiviral vectors, determination of viral RNA content, and infection were performed as previously described ([Torisu *et al.* 2013](#_ENREF_5)).

**Protein Expression Analysis**

HUVECs were transfected with ATG7 siRNA or a scrambled control siRNA for 24 hours and then treated with oxLDL (50 μg/ml) or LDL (Alfa Aesar; 50 μg/ml) for additional 24 hours. After 24 hours of treatment, total protein was extracted using RIPA buffer (Sigma). Levels of protein expression were obtained by Western blotting using antibodies directed against Atg7, p62, LC3 (all from Cell Signaling), LDLr (Santa Cruz) and GAPDH (Millipore). Following incubation with the appropriate horseradish peroxidase-associated secondary antibodies (Santa Cruz Biotechnology), bands were visualized with an enhanced chemiluminescence detection system (Amersham/GE Healthcare Biosciences), and their intensities quantified by densitometry using the ImageJ software. LC3 post-translational modification is represented as the ratio of LC3-II/I expression.

**Electron microscopy:**

For analysis of autophagosome formation following exposure to OxLDL (50 μg/ml for 24 hours), endothelial cells were fixed (2% glutaraldehyde in cacodylate buffer, pH 7.3) for at least 2 h at 4°C and then post-fixed in 1% osmium tetroxide before embedding in Quetol-Spurr resin and polymerizing at 65^0^ C overnight. The sections were examined with a FEI Tecnai Transmission Electron Microscope equipped with a digital image acquisition system.

For analysis of native LDL incorporation into autophagosomal structures, native LDL cholesterol (KALEN Biotechnologies) was labeled with a 10 nm gold nanoparticle conjugation optimization kit according to the manufacture’s protocol (Cytodiagnostics). After gold labeling, unconjugated gold particles were removed using a Sephadex G50 column (GE Healthcare). HUVECs were plated onto 2-well Labtek Chamber Permanox slide (Thermo Scientific) in endothelial growth medium-2 media (EGM-2; Lonza) containing 10% lipoprotein-deficient serum (KALEN Biotechnologies). Gold-labeled LDL was incubated with cells for 24 hours after which HUVECs were fixed, silver-enhanced and imaged as previously described ([Torisu *et al.* 2013](#_ENREF_5)).

**Native and oxidized LDL uptake and live cell image analysis:**

Fluorescence of living cells was performed using Zeiss LSM 510 confocal microscope (Carl Zeiss MicroImaging) using the 63× NA 1.4 oil-immersion and a 488 nm laser excitation and 505-550 nm emission filter for GFP and 594 nm laser excitation and a LP 610 nm emission filter for diI-labeled lipids. For quantification of OxLDL uptake, cells were incubated with dil-OxLDL (50 μg/ml) for 24h and then thoroughly washed to remove non-incorporated fluorescent lipids. To measure amounts of diI-OxLDL accumulated in cells, intracellular diI-OxLDL was extracted with isopropanol, and the fluorescence counted in SpectraMax M5 (Molecular Devices).

For measurement of LDL transcytosis, HUVECs were plated onto fibronectin and gelatin-coated trans- well inserts with a pore size of 0.4 μm (Millicell-CM, Millipore) in 12-well multiwell plate and cultured in EGM-2 containing 10% lipoprotein-deficient serum for 24 hours. The integrity of HUVECs monolayer was validated by low paracellular transport of inulin-FITC (Sigma), with the transfer of inulin-FITC to the lower compartment usually less than 5% of upper compartment. We added 5 μg of ^125^I-LDL cholesterol to the upper chamber and after a 24 hour incubation, samples were taken from the upper and lower compartments. Radioactive samples were counted using a Wallac 1450 MicroBeta TriLux liquid scintillation counter (Perkin Elmer). Culture media was concentrated using trichloroacetic acid (Sigma) precipitation before measurement. For measurement of cell surface or intracellular accumulation of LDL cholesterol, cells were incubated in 10 mg/mL heparin (Sigma) at 4^0^C for 1 hour to remove cell-surface binding of ^125^I-LDL cholesterol as previously described ([Vlodavsky *et al.* 1978](#_ENREF_6)). Heparin-digested ^125^I-LDL cholesterol was used to determine surface binding. Cells were then lysed in 0.1 N NaOH. Prior to scintillation counting, the cell lysates were neutralized with 10% acetic acid (Sigma). We noted that lentiviral infection resulted in a modest effect on ^125^I-LDL cholesterol accumulation (compare 1G and 1H).

**Immunofluorescence and LC3 dot quantification**

HUVECs were transfected with either siAtg7 or scrambled control for 24 hours and then treated with Dil-OxLDL (50 µg/ml for 24 hours). Immunofluorescent signals from Lamp-1 and perilipin (both Santa Cruz) staining were visualized with standard protocols. Images were acquired using Zeiss LSM700 confocal microscope and processed using ZEN imaging software. LC dots were quantified after electroporation of HUVECs with a GFP-LC3. After 24 hours of electroporation, cells were pre-treated with chloroquine for 2 hours before incubation with OxLDL (50 µg/ml) for additional 12 hours. Cells were then fixed for confocal microscopy. In the other group, lentiviral shAtg7- and control-transfected HUVECs were electroporated with GFP-LC3 plasmid. After 24 hours of electroporation, HUVECs were treated with OxLDL (50 µg/ml) for 24 hours. Cells were then fixed for confocal microscopy. Nuclei were stained with DAPI. Numbers of green dots per cell were quantified using Zeiss LSM700 confocal microscope.

**Retinal lipid deposition**

To evaluate OxLDL uptake by retinal pigment epithelial (RPE) and choriocapillary endothelial cells, Atg7^endo^ (fl/fl;VE-Cadherin Cre) and WT (WT/WT; VE-Cadherin Cre ) mice were intravenously injected using 10 μg/g DiI-OxLDL (Biomedical Technology Inc). Mice were euthanatized 24 and 48 hours after injection; eyes were collected and fixed in 4% paraformaldehyde in PBS for 2 hours. The tissue was then embedded in OCT (Tissue Tek) and cut into thin sections (10μm). Nuclei were labeled with TO-PRO-3 (1:1000, Life Technologies) and retinal epithelial cells with Glut1 (Abcam). Sections were mounted with Dako fluorescent mounting medium. Fluorescence was observed with a confocal microscope in the mouse RPE and choriocapillaris/choroid cells beneath the RPE. Six eyes were analyzed per group, with 5 sections per eye and 6 frames per section at 800X magnification (180 events per group). Image analysis was carried out using Image J version 1.48e. The region of interest (ROI) was selected in white light transmission channel to include the RPE and the underlying choriocapillaris/choroid. The ROI was then evaluated in the DiI-OxLDL fluorescence (red) channel and threshold set to minimum 25 and maximum 255 to capture the number of pixels that display fluorescence. Images were quantified as percentage (%) of fluorescent pixels within the ROI.

**Atherosclerosis analysis:**

Male mice were weaned at 3 weeks of age and fed standard rodent chow for 1 week, and were then changed to a high fat diet (standard diet- 23% protein, 22 % fat and 55% carbohydrates; high fat diet- 15% protein, 42% fat, 43 % carbohydrate). Mice were fed the high fat diet for 4, 12, and 20 weeks, from 4 to 8, 4 to 20, and 4 to 24 weeks of age respectively. We used only male mice for the 8 week time point and only female mice for the 16 and 24 week time points. The weight of the mice was monitored weekly and body composition was determined using a MiniSpec NMR analyzer (Bruker).

For en face analysis, aortic tissue (including the proximal aorta and both iliac arteries) were stained with Oil Red O. The percentage of the stained area was calculated from images acquired using a Leica fluorescence stereo-microscope (Leica microsystems). To determine the cross-sectional lesion area, serial cross-sections (8 µm) of aortic root were prepared with a cryostat according to the method previously described ([Ni *et al.* 2003](#_ENREF_3)). In brief, six levels within the aortic sinus region were analyzed. These levels were separated by 120 µm with the most proximal site beginning where the three aortic valves first appear. Using EVG-stained or Oil red O-stained sections, the atherosclerotic area was quantified. Five levels within the aortic arch, separated by 120 µm, were quantified. Light field images were captured using Nikon wide field fluorescent microscope equipped with digital camera. All images were analyzed using Photoshop 7.0 (Adobe) and Fiji software.

For histochemical analysis, mouse tissues were fixed with 4% PFA overnight and then cryoprotected by 10% sucrose in PBS followed by 20% sucrose in PBS over 48 h at 4°C. Tissues were then embedded in OCT compound (Sakura Finetek, Japan), and snap-frozen in liquid nitrogen and stored at −80°C. Immunohistochemistry was performed with a rat monoclonal anti-MOMA-2 (BMA Biomedicals), mouse monoclonal CD31 (Abcam) antibody or Masson’s Trichrome staining according to standard methods. The same region near the aortic valve was quantified for plaque composition. Necrotic area was quantified by tracing acellular areas using hematoxylen and eosin stained sections. Light field or fluorescent images were captured using Nikon wide field fluorescent microscope (Nikon) equipped with digital camera. All images were analyzed using Photoshop 7.0 (Adobe) and Fiji software.

**Serum metabolic profile:**

Blood glucose was determined using a one-touch Ascensia Elite glucometer (Fischer). Serum lipoproteins (VLDL, LDL and HDL) were separated using low pressure gel filtration on a Superose 6 Increase column: 10/300GL (GE Healthcare). Elution buffer was comprised of 10 mM Tris/HCl, 0.15M NaCl, 0.02% sodium azide, 2 mM CaCl_2_, 100µM DTPA, pH 7.4, at a flowrate 0.5ml/min. Typically, 100µl serum was injected onto the column.  After 12 mins, fractions were collected every minute for a total of 25 fractions.  These were manually assayed for cholesterol using the Beckman Coulter Synchron reagent. A cholesterol standard curve was generated using an in-house serum calibrator. Triglycerides were manually assayed using the Beckman Coulter Synchron reagent. A Triglyceride standard curve was generated using an in-house serum calibrator. Serum free fatty acids were determined by using a commercial assay system (WAKO).

**Supplemental References:**

Komatsu M, Waguri S, Ueno T, Iwata J, Murata S, Tanida I, Ezaki J, Mizushima N, Ohsumi Y, Uchiyama Y, Kominami E, Tanaka K, Chiba T (2005). Impairment of starvation-induced and constitutive autophagy in Atg7-deficient mice. *J Cell Biol*. **169**, 425-434.

Lee IH, Cao L, Mostoslavsky R, Lombard DB, Liu J, Bruns NE, Tsokos M, Alt FW, Finkel T (2008). A role for the NAD-dependent deacetylase Sirt1 in the regulation of autophagy. *Proc Natl Acad Sci U S A*. **105**, 3374-3379.

Ni W, Tsuda Y, Takashima S, Sato H, Sato M, Imaizumi K (2003). Anti-atherogenic effect of soya and rice-protein isolate, compared with casein, in apolipoprotein E-deficient mice. *Br J Nutr*. **90**, 13-20.

Singh KK, Lovren F, Pan Y, Quan A, Ramadan A, Matkar PN, Ehsan M, Sandhu P, Mantella LE, Gupta N, Teoh H, Parotto M, Tabuchi A, Kuebler WM, Al-Omran M, Finkel T, Verma S (2015). The Essential Autophagy Gene ATG7 Modulates Organ Fibrosis via Regulation of Endothelial-to-Mesenchymal Transition. *J Biol Chem*. **290**, 2547-2559.

Torisu T, Torisu K, Lee IH, Liu J, Malide D, Combs CA, Wu XS, Rovira, II, Fergusson MM, Weigert R, Connelly PS, Daniels MP, Komatsu M, Cao L, Finkel T (2013). Autophagy regulates endothelial cell processing, maturation and secretion of von Willebrand factor. *Nat Med*. **19**, 1281-1287.

Vlodavsky I, Fielding PE, Fielding CJ, Gospodarowicz D (1978). Role of contact inhibition in the regulation of receptor-mediated uptake of low density lipoprotein in cultured vascular endothelial cells. *Proc Natl Acad Sci U S A*. **75**, 356-360.
