# Supplementary material for: Intact endothelial autophagy is required to maintain vascular lipid homeostasis
Source: Aging Cell. 2015 Nov 24;15(1):187–91. doi: 10.1111/acel.12423 (PMC4717267; doi:10.1111/acel.12423)
Supplement: Supplementary file 3 [file ACEL-15-187-s003.docx]

**Supplemental Figure Legends**

**Figure 1**: Characterization of the role of autophagy in endothelial lipid homeostasis. A) Time course of knockdown following transfection of siRNA directed at ATG7 in HUVECs. Levels of Atg7 are shown by Western blot. In addition, as a marker of autophagic flux, the conversion of LC3I (top band) to LC3II (bottom band) is shown as is p62 and GAPDH as a loading control. B) Quantification of the LC3-II/LC3-I ratio as a biochemical readout of autophagic flux following ATG7 knockdown (n=4 per group). C) Quantification of p62 levels as a measure of autophagic flux (n=4-6 per group). D) HUVECs were electroporated with a LC3-GFP plasmid. One day after transfection, cells, where indicated, were treated with chloroquine (CQ) for 2 hours before incubation with ox-LDL (50 µg/ml) for an additional 12 hours. Cells were then fixed and nuclei were stained with DAPI (blue). n>25 random cells per condition. E) LC3-GFP fluorescence in HUVECs previously infected with either a control shRNA or an shRNA directed against ATG7 and exposed, where indicated, to ox-LDL (50 μg/ml) for 24 hours. The number of green punctae per cell was quantified, with greater than 25 cells per condition analyzed. F) Western blot for LC3, p62 and GAPDH on protein lysates isolated from ATG7 silenced (siAtg7) HUVECs or scrambled control (Scr)-transfected cells. Analysis was performed 24 hours following LDL (50 µg/ml) treatment. G) Quantification of the LC3-II/LC3-I ratio (n=6) for LDL-stimulated autophagosome formation. H) Confocal image of HUVECs transfected with a GFP-LC3 plasmid (green) and exposed to fluorescently labeled DiI-LDL (red). The lipid, in some cases, appears to be surrounded by circular LC3-coated structures, consistent with an autophagosome . I) Western blot for total LDL receptor levels or in control or Atg7 siRNA knockdown cells. GAPDH is shown as a loading control. J) Surface binding of ^125^I-LDL cholesterol to control or Atg7 shRNA knockdown cells. Binding was assessed after 4-5 hour incubation with ^125^I-LDL cholesterol. Cells were incubated in 10 mg/mL heparin at 4^0^C for 1 hour to determine cell-surface binding of ^125^I-LDL cholesterol. Average of n=3 separate experiments is shown. p=NS between groups. K) Left, immunofluorescence overlay of perilipin (green), an indication of neutral lipid stores, and DiI-OxLDL (red). Similar overlay on the right of the lysosomal marker Lamp1 (green) with DiI-OxLDL (red). Nuclei were stained with DAPI (blue). Some degree of overlap is seen in both conditions. Where there are multiple groups, p values was obtained by performing a Bonferroni post hoc test after a one-way ANOVA.

**Figure 2:** Characterization of plaque composition. Representative sections of aortic cross sections stained for A) a macrophage marker (MOMA-2). B) Masson’s Trichrome for collagen deposition (blue) and C) an endothelial marker (CD31). D) Quantification of MOMA- staining (n=5 ApoE KO mice and n=4 Atg7^endo^/ApoE KO). E) Level of fibrosis as assessed by Masson Trichrome staining (n=3 ApoE KO mice and n=3 Atg7^endo^/ApoE KO mice . F) Necrotic area in control (n=4 ApoE KO mice) and experimental (n=3 Atg7^endo^/ApoE KO) mice. While the trend was for increased fibrotic and necrotic area in the Atg7^endo^/ApoE KO animals, no relationship reached significance, most likely due to the limited sample size.

**Table 1**: Comparison of serum and metabolic parameters between ApoE KO (WT/WT VE-cadherin Cre/ApoE^-/-^) and Atg7^endo^/ApoE KO mice (fl/fl VE-Cadherin Cre/ApoE^-/-^). Parameters measured include: total cholesterol, triacylglycerol, LDL cholesterol, HDL cholesterol, free fatty acid, fasting glucose, body weight and fat composition. The number of animals per group is shown and varied depending on the test.
